# The relationship between ovarian function and ovarian limited dose in radiotherapy postoperation of ovarian transposition in young patients with cervical cancer

**DOI:** 10.1002/cam4.924

**Published:** 2017-02-17

**Authors:** Zhenhua Du, Hui Qu

**Affiliations:** ^1^ Department of obstetrics and gynecologyShengJing hospital of China medical universityShenyangLiaoning110022China

**Keywords:** Early cervical cancer, ovarian function, ovarian limited dose in radiotherapy, ovarian transposition

## Abstract

In this study, the relationship between ovarian function and ovarian limited dose in radiotherapy was evaluated in young patients with cervical cancer who underwent ovarian transposition (Fig1B). Moreover, the novel ovarian dose limit for a better preservation of ovarian function in intensity‐modulated radiation therapy (IMRT) was determined. We retrospectively analyzed data from 86 patients with cervical cancer who received radical hysterectomy and ovarian transposition from January 2013 to June 2015. In agreement with the National Comprehensive Cancer Network Guidelines (NCCN) for Cervical Cancer Version 2.2015, 65 patients with pathological high‐risk factors were administered adjuvant radiotherapy—20 of them received three‐dimensional conformal radiotherapy (Observation Group A), 24 patients received IMRT with no limitation on radiation dose to ovaries (Observation Group B), and 21 patients underwent IMRT with limited radiation dose(V_10_<20%) to ovaries (Observation Group C). Twenty‐one patients without any predetermined high‐risk factors did not received radiation therapy (Control Group D). Patients from all four groups were followed up, and sex hormone levels (E_2_, P, follicle‐stimulating hormone [FSH], LH) before radiation, postradiation, 3 month, and 6 month after the radiation therapy were measured by electrochemiluminescence immunoassay. Subsequently, changes in sex hormone levels in all four groups of patients at various time points were analyzed. The levels of sexual hormones (E_2_, P, FSH, LH) before radiation, postradiation, 3 month, and 6 month after the radiation therapy in patients from all three observation groups were significantly lower than those in patients of the control group (*P* < 0.05). There was no statistically significant difference in the levels of sex hormones in patients of the control group at different time points (*P* > 0.05). Within each observation group, there was a statistically significant difference in the sex hormone levels in patients before the radiation and after the radiation (*P *<* *0.05); however, when data from all three observation groups were compared, only the difference in the levels of FSH and LH between the patients from Group A and Group C was statistically significant (*P *<* *0.05). The results of receiver‐operating characteristic (ROC) curve analysis suggested that limiting ovarian radiation dose to V_7.5 _< 26% in IMRT prevents the disruption of ovarian function (area under ROC curve was 0.740, confidence interval [CI] = 0.606–0.874). In young patients with cervical cancer who underwent radical hysterectomy and ovarian transposition without receiving adjuvant radiotherapy, ovarian endocrine function was well preserved. In patients who received any type of postoperative radiotherapy, ovarian function was affected, suggesting that the standard ovarian limited dose used in IMRT disrupted ovarian function. The results of the ROC curve analysis suggested that the new optimal dose limit of V_7.5_ < 26% should be used in IMRT to preserve ovarian function (*P *=* *0.003).

## Introduction

Cervical cancer is one of the most common gynecologic malignancies in China and is the second common female malignant tumor worldwide. There was a significant increase in incidence of cervical cancer in the recent years with a new trend of it disproportionally affecting younger population that is believed to be associated with the changes in the standards of living and lifestyle 1. Furthermore, the widespread use of gynecological diseases census, cervical cytological examination, colposcopy etc raised the rates of early diagnosis. The cure rates of patients with the early stages of cervical carcinoma are high, and therefore, preserving ovarian function is a vital quality of life factor for those young patients. Currently, patients with the early stages of cervical cancer undergo radical hysterectomy and ovarian transposition to preserve ovarian function. National Comprehensive Cancer Network (NCCN) Guidelines for Cervical Cancer Version 2.2015, recommend postoperative adjuvant radiotherapy for patients with high‐risk pathological factors. It has been reported that premenopausal hysterectomy can accelerate ovarian failure 2. Moreover, ovaries are very sensitive to radiation that can lead to ovarian failure, different symptoms of the surgery‐induced menopause, and endocrine dyscrasia. The degree of ovarian damage varies depending on the limited dose of radiation received by ovaries. Here, we aim to test whether the standard limited ovarian dose of V_10_ < 20% for single or both ovary delivered by intensity‐modulated radiation therapy (IMRT) is able to preserve ovarian function 3, and if this is not the case, to determine dose that would not impede ovarian function. Therefore, we performed clinical studies to determine the radiation dose limit that can preserve ovarian function. In this study, 86 young patients with stages Ib or IIa cervical cancer, who underwent radical hysterectomy and ovarian transposition, were divided into four groups (A, B, C, and control group D) depending on the presence and the type of radiation therapy. Serum level of sex hormones (E_2_, P, follicle‐stimulating hormone [FSH], LH) was used to evaluate the relationship between ovarian function and ovarian radiation dose in young patients with cervical cancer who underwent ovarian transposition. Furthermore, we attempted to find the optimal radiation dose limit to preserve ovarian function in IMRT.

## Materials and Methods

### Study design

#### Epidemiologic data

##### Inclusive criteria

(1) Histopathological diagnosis of cervical cancer;(2) clinical stage Ib–IIa, with treatment of surgery, unilateral or bilateral reservation and suspension of ovary;(3) age: 25–40 years old;(4) KPS≥70;(5) without climacteric symptoms such as hectic fever, night sweating, and insomnia;(6) no hormone replace therapy.

##### Exclusive criteria

(1) Those who do not meet the criteria above;(2) quitted during the radiotherapy treatment;(3) there were serious adverse reactions during radiotherapy, such as infection, fever, bone marrow suppression, extend the time of radiotherapy.

According to the criteria above, we retrospectively analyzed 86 cases of cervical cancer in patients who were admitted to Shengjing Hospital of China Medical University from January 2013 to June 2015. The age of the patients ranged from 26 to 40 years, and the median age was 35 years. While at the hospital, patients underwent radical hysterectomy and ovarian transposition. Clinical staging of the tumors, which was done according to the International Federation of Gynecology and Obstetrics (FIGO) 2009 Criteria 4, identified 23 cases of I b_1_ stage, 28 cases of I b_2_, 18 cases of II a_1_, and 17 cases of II a_2_. Histological examination identified 62 cases of squamous cell carcinoma, 16 cases of adenocarcinoma, and eight cases of adenosquamous carcinoma. Ovarian transposition was performed on one ovary in 13 cases and on both ovaries in 73 cases. Sixty‐five patients with high‐risk pathological factors received adjuvant radiotherapy according to the NCCN guidelines: 20 patients received 3D conformal radiotherapy (Observation Group A), 24 patients received IMRT with no limit on radiation dose to ovaries (Observation Group B), and 21 patients received IMRT with limited radiation dose V_10_<20% (Observation Group C). Twenty‐one patients with no predetermined high‐risk factors did not receive radiation therapy (Control Group). The radiation dose was set as DT4500–5000 cGy /25–28f/5w. Details are given in Table 1. Authors had access to identifying information during data collection.

**Table 1:** Patients’ characteristics

Group	A (%) *n* = 20	B (%) *n* = 24	C (%) *n* = 21	Control (%) *n* = 21	*P*
Ag**e (**Range)	33 (26–38)	35 (27–40)	36 (26–39)	35 (26–40)	0.98
FIGO staging					
Ib1	4 (20.0)	8 (33.3)	6 (28.6)	5 (23.8)	0.77
Ib2	6 (30.0)	9 (37.5)	7 (33.3)	6 (28.6)	0.89
IIa1	5 (25.0)	4 (16.7)	4 (19.0)	5 (23.8)	0.89
IIa2	5 (25.0)	3 (12.5)	4 (19.0)	5 (23.8)	0.71
Histological types					
Squamous cell carcinoma	15 (75.0)	18 (75.0)	14 (66.7)	15 (71.4)	0.91
Adenocarcinoma	2 (10.0)	4 (16.7)	6 (28.6)	4 (19.0)	0.49
Adenosquamous carcinoma	3 (15.0)	2 (8.3)	1 (4.8)	2 (9.5)	0.72
Surgical procedures					
One ovarian transposition	3 (15.0)	4 (16.7)	3 (14.3)	3 (14.3)	0.99
Ovarian transposition	17 (85.0)	20 (83.3)	18 (85.7)	18 (85.7)	0.99
Concurrent chemotherapy	16 (80.0)	19 (79.0)	17 (81.0)	0 (0.0)	0.99
Pelvic radiation dose	4500–5000 cGy	4500–5000 cGy	4500–5000 cGy	4500–5000 cGy	
Ovary limit dose	No	No	V_10_ < 20%	No	
Brachytherapy	12 (60.0)	13 (54.2)	11 (52.4)	12 (57.1)	0.93

There is no statistically significant difference at age and clinical staging among four groups (*P* < 0.05). Because the chemotherapy was not used in control group, we only compared the experimental group combined with chemotherapy.

### Treatment methods

#### Surgical techniques

**Figure 1:**
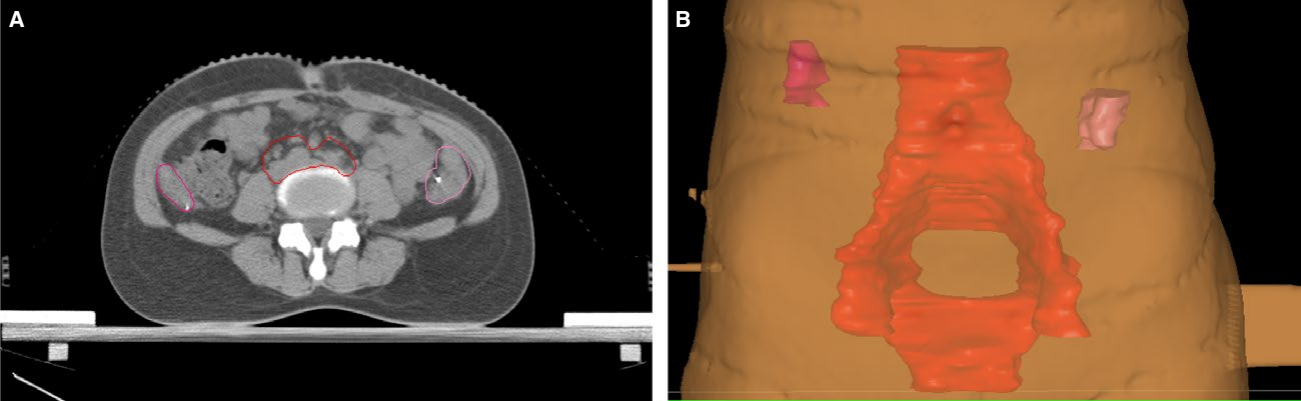
Ovary position: (A) ovary position in CT image; (B) reconstruction of ovary position in IMRT plan system. Red region is the patient's clinical target volume (CTV). IMRT, intensity‐modulated radiation therapy.

All 86 patients received radical hysterectomy, pelvic lymphadenectomy, and ovarian transposition (patients younger than 45 years old, the ovarian appearance was normal and no abnormalities in intraoperative frozen pathological exam and sign in ovarian transposition informed consent, then ovary transposition would be used), which included 13 cases of one ovary transposition and 73 cases of both ovaries transposition. Surgery was performed according to the following scheme: laparotomy was followed by pelvic lymphadenectomy; next, the serosa was opened by cutting along infundibulopelvic ligament ovarian vessel, ovarian vein, and artery were isolated up to the bifurcation of common iliac artery, ovarian ligament, and isthmic portion were cut off, ovaries were inspected and, if normal, were covered with gauze saturated with sterile physiological saline. Next, patients underwent extensive total hysterectomy, ovaries were moved more than 2 cm above iliac crest and under parietal peritoneum (equivalent to horizontal of the forth lumbar vertebrae) and secured with silver clips on both the top and the bottom for labeling (localization of radiation therapy)(Fig. 1). If no twisting of ovarian vessel was detected, and vascular tension, as well as blood circulation, was normal, the conventional abdominal operation was performed.

#### Radiation treatment

According to the NCCN guidelines, pathological examination after surgery was conducted to evaluate the following recurrence (high‐risk) factors: positive lymph nodes, positive resection margin, positive cervical tissue, vascular involvement, tumor invasion of cervical stroma, and local lesions larger than 4 cm. Patients with predetermined high‐risk factors were treated with radiation therapy 2–4 weeks after the surgery. Two different radiation techniques were used: (1) Four‐field bilateral three‐dimensional conformal radiotherapy (3DCRT) with radiation emitted in the forward and backward directions, with dose of target (DT) 4500– 5000 Gy/25–28f/5w and (2) intensity‐modulated radiation therapy (IMRT) with the CT‐localized body membranes of patients outlining clinical target volume (CTV) in treatment plan system (TPS) and 95% of isodose curve covering CTV. Planning target volume (PTV) was defined as CTV plus 0.8 cm margin. CTV included vaginal stump, presacral and paraneoplastic areas, lymphatic drainage area of iliac vasculature and organs determined to be at risk (rectum, bladder, colorectal, intestine, or femoral head). The upper boundary was located at L4–L5, and the lower boundary was located at the inferior margin of pubic symphysis, which properly denotes downward invasion of vagina. Bilateral boundary was located on the sidewall of the basin. The dose of CTV was DT4500‐5000 cGy /25‐28f/5w, and the dose of organs at risks: Rectal V_45_ ≤ 50%, bladder V_45_ ≤ 50%, large intestine V40 ≤ 40%, small intestine V40≤ 25%, V45 ≤ 14%, femoral head V40 ≤ 5%, ovarian V_10_ ≤ 20%. For the patients at Ib2 and IIa2 stage, before surgery, cervical lesions>4 cm or pathological lymph node metastasis or vaginal margin, parametrial (+), according to the NCCN guidelines, intracavitary radiotherapy was adopted at the end of external beam radiation therapy. Vaginal cuff end submucosal 0.5 cm DT 600 cGy /5f/3w,2f/w. The dose limit to the ovary in IMRT was V_10_ < 20%.

#### Adjuvant chemotherapy

In 52 cases, adjuvant concurrently chemotherapy included 25 mg/m^2^ of cisplatin administered weekly for 4–6 weeks.^5^.

#### Follow‐up index

The fasting blood levels of sex hormones—estrogen (E_2_, pmol/L), progestin (P, nmol/L), follicle‐stimulating hormone (FSH, IU/L), and luteinizing hormone (LH, IU/L)—were evaluated in all patients before radiation, postradiation, 3 month, and 6 month after the radiation therapy. All patients were followed up for 6 month, and no clinical recurrence was detected.

### Statistical analysis

A significant difference in the levels of sex hormones was detected in patients before radiation, postradiation, 3 month, and 6 month after radiation therapy. The results are presented as mean ± SD. A t‐test and confidence interval (95% CI) analysis was conducted using SPSS19.0 software. Calculation of the optimal limited radiation dose that would preserve ovarian function was based on the receiver‐operating characteristic (ROC) curve analysis of the relationship between the radiation dose and the volume of exposure to radiation.

## Results

### Comparison of sex hormone levels in serum of patients of the experimental and control groups

While there was no significant difference in sex hormones (E_2,_ P, FSH, LH) serum levels between patients in the treated and control groups before the radiation (*P* > 0.05), serum levels of hormones in patients from the experimental groups were significantly different (*P* < 0.05) from those in patients of the control group at postradiation, 3 month, and 6 month time points (Table 2).

**Table 2:** Sex hormone levels in all patient groups at different time points

		E2	*P*_E2_	P	*P*_P_	FSH	*P*_FSH_	LH	*P*_LH_
Pre radiation	CK	85.80 ± 74.51	1 (Ref.)	2.73 ± 4.71	1 (Ref.)	28.74 ± 37.44	1 (Ref.)	16.98 ± 20.40	**1 (Ref.)**
	A	85.80 ± 74.51	0.505	2.73 ± 4.71	0.710	28.74 ± 37.44	0.053	16.98 ± 20.40	**0.051**
	B	165.17 ± 151.84	0.295	2.19 ± 3.89	0.508	16.15 ± 29.29	0.579	15.78 ± 20.65	**0.106**
	C	103.50 ± 68.14	0.534	3.38 ± 3.64	0.156	10.98 ± 11.36	0.285	27.02 ± 19.76	**0.679**
Postradiation	CK	156.30 ± 85.59	1 (Ref.)	2.73 ± 4.71	1 (Ref.)	8.60 ± 6.55	1 (Ref.)	10.79 ± 10.59	1 (Ref.)
	A	30.80 ± 20.12	0.002	0.58 ± 0.35	<0.001	81.77 ± 27.11	<0.001	51.82 ± 17.63	**0.036**
	B	35.42 ± 18.32	**<0.001**	1.64 ± 0.65	0.015	73.57 ± 27.58	**<0.001**	62.73 ± 19.37	**<0.001**
	C	46.70 ± 37.12	0.002	0.68 ± 0.19	0.026	48.70 ± 32.16	0.001	39.26 ± 13.30	**0.034**
3 month after	CK	101.60 ± 83.45	1 (Ref.)	4.42 ± 4.89	1 (Ref.)	14.90 ± 16.35	1 (Ref.)	12.40 ± 11.56	1 (Ref.)
	A	32.00 ± 16.54	0.008	1.06 ± 0.46	0.021	97.33 ± 23.29	<0.001	59.15 ± 15.20	**<0.001**
	B	30.58 ± 8.00	0.003	0.84 ± 0.48	0.021	90.78 ± 31.54	**<0.001**	54.23 ± 19.90	**<0.001**
	C	32.90 ± 18.86	0.003	0.49 ± 0.25	0.004	70.19 ± 30.5	0.001	45.75 ± 20.71	**0.005**
6 month after	CK	99.40 ± 68.11	1 (Ref.)	4.74 ± 5.25	1 (Ref.)	17.68 ± 22.29	1 (Ref.)	17.64 ± 16.39	1 (Ref.)
	A	29.00 ± 14.73	0.005	0.49 ± 0.19	0.020	88.11 ± 34.43	**<0.001**	56.32 ± 24.68	**0.001**
	B	26.50 ± 10.88	0.002	0.56 ± 0.38	0.012	106.44 ± 37.18	**<0.001**	64.12 ± 16.27	**0.001**
	C	29.80 ± 7.51	0.005	0.47 ± 0.32	0.019	77.81 ± 31.94	**<0.001**	54.76 ± 24.99	**0.001**

E2, estrogen; P, progestin; FHS, follicle‐stimulating hormone; LH, luteinizing hormone; Ref., reference.

### Comparison of serum sex hormone levels in patients of the experimental groups before and after radiation

**Figure 2:**
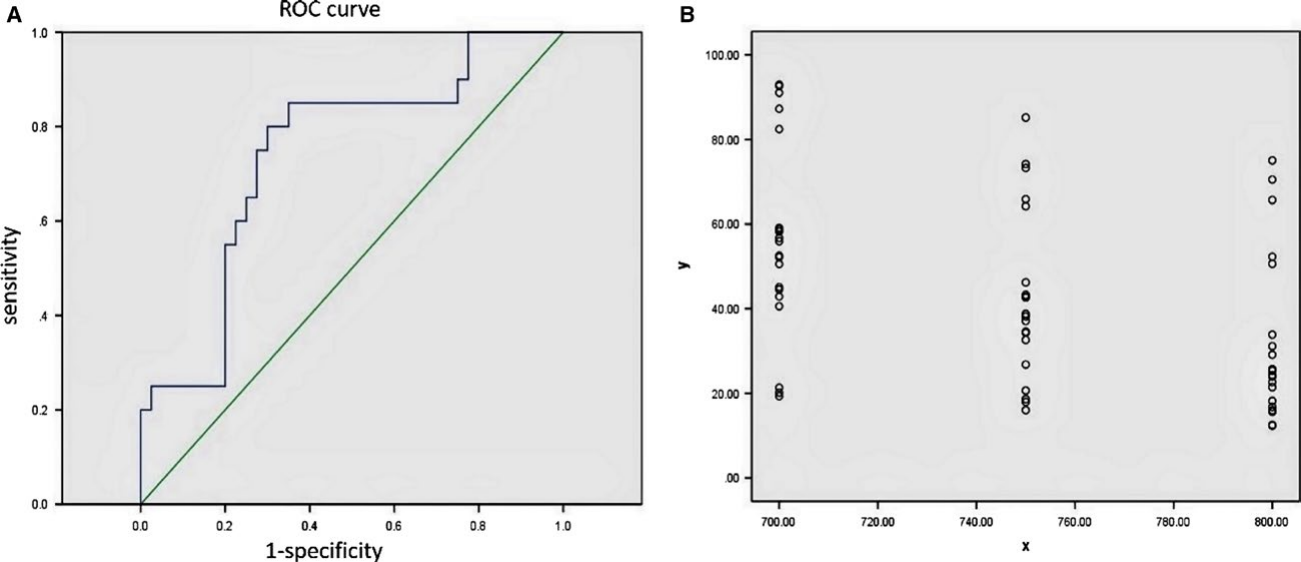
Receiver‐operating characteristic (ROC) Curve statistics and dose–volume histograms of 21 patients receiving intensity‐modulated radiation therapy (IMRT). (A) ROC Curve statistics for determining the optimal ovarian limited dose. Area under the curve (AUC) is 0.740 (95% CI = 0.606–0.874), *P* = 0.003. (B) Using Photoshop software, linear superposition method was applied to the ovarian radiation doses of 21 patients who received IMRT, and the results revealed the range of 700–800 cGy. Dose–volume histograms were created with radiation doses of 700–800 cGy as abscissa and ovarian radiation volumes as ordinate.

There was a significant difference in sex hormone levels between patients of the experimental groups both before and after the radiation (Table 3, P < 0.05). Patients in Group A had significantly higher levels of FSH and LH *(P* < 0.05), than patients in Group C (Table 4). Using area under the ROC curve, and 95% CI statistical analysis, we determined that the optimal limited radiation dose well tolerated by ovaries was V_7.5_ < 26% (Fig. 2). The area under the curve was 0.740, and the 95% CI was 0.606–0.874. Levels of hormones right after irradiation, 3 month or 6 month after the radiation therapy were not significantly different (*P* > 0.05). Therefore, we concluded that there was no recovery or further decline of ovarian function 6 month after the radiation (Table 3).

**Table 3:** Sex hormone levels in patients of observation and control groups at different times

		E2	*P*_E2_	*P*_E2_	P	*P*_P_	*P*_P_	FSH	*P*_FSH_	*P*_FSH_	LH	*P*_LH_	*P*_LH_
A	Preradiation	85.80 ± 74.51	**1 (Ref.)**		2.73 ± 4.71	**1 (Ref.)**		28.74 ± 37.44	**1 (Ref.)**		16.98 ± 20.40	**1 (Ref.)**	
	Postradiation	30.80 ± 20.12	0.039	**1 (Ref.)**	0.58 ± 0.35	0.032	1 (Ref.)	81.77 ± 27.11	<0.001	**1 (Ref.)**	51.82 ± 17.63	0.001	**1 (Ref.)**
	3 month after	32 ± 16.54	0.008	0.819	1.06 ± 0.46	0.021	0.819	97.33 ± 23.29	<0.001	0.141	59.15 ± 15.20	<0.001	0.222
	6 month after	29 ± 14.73	0.005	0.636	0.49 ± 0.19	0.02	0.636	88.11 ± 34.43	<0.001	0.671	56.32 ± 24.68	0.001	0.609
B	Preradiation	165.17 ± 151.84	**1 (Ref.)**		2.19 ± 3.89	**1 (Ref.)**		16.15 ± 29.29	**1 (Ref.)**		15.78 ± 20.65	**1 (Ref.)**	
	Postradiation	35.42 ± 18.32	0.017	**1 (Ref.)**	1.64 ± 0.65	0.042	**1 (Ref.)**	73.57 ± 27.58	<0.001	**1 (Ref.)**	62.73 ± 19.37	0.001	**1 (Ref.)**
	3 month after	30.58 ± 8.00	0.003	0.384	0.84 ± 0.48	0.021	0.384	90.78 ± 31.54	<0.001	0.204	59.15 ± 15.20	<0.001	0.365
	6 month after	26.50 ± 10.88	0.002	0.149	0.56 ± 0.38	0.012	0.149	106.44 ± 37.18	<0.001	0.15	54.23 ± 19.90	<0.001	0.08
C	Preradiation	103.5 ± 68.14	**1 (Ref.)**		3.38 ± 3.64	**1 (Ref.)**		10.98 ± 11.36	**1 (Ref.)**		27.02 ± 19.76	**1 (Ref.)**	
	Postradiation	46.7 ± 37.12	0.003	**1 (Ref.)**	0.68 ± 0.19	0.045	**1 (Ref.)**	48.70 ± 32.16	<0.001	**1 (Ref.)**	39.26 ± 13.3	0.008	**1 (Ref.)**
	3 month after	32.90 ± 18.86	0.003	0.301	0.49 ± 0.25	0.004	0.867	70.19 ± 30.5	0.001	0.25	45.75 ± 20.71	0.005	0.406
	6 month after	29.80 ± 7.51	0.005	0.142	0.47 ± 0.32	0.019	0.659	77.81 ± 31.94	<0.001	0.053	54.76 ± 24.99	0.001	0.375
Control	Preradiation	85.8 ± 74.51	**1 (Ref.)**		2.73 ± 4.71	**1 (Ref.)**		28.74 ± 37.44	**1 (Ref.)**		16.98 ± 20.40	**1 (Ref.)**	
	Postradiation	156.3 ± 85.59	0.225		2.73 ± 4.71	0.143		8.6 ± 6.55	0.689		10.79 ± 10.59	0.874	
	3 month after	101.6 ± 83.45	0.085		4.42 ± 4.89	0.152		14.9 ± 16.35	0.588		12.4 ± 11.56	0.651	
	6 month after	99.4 ± 68.11	0.686		4.74 ± 5.25	0.108		17.68 ± 22.29	0.344		17.64 ± 16.39	0.108	

E2, estrogen; P, progestin; FSH, follicle‐stimulating hormone; LH, luteinizing hormone; Ref., reference.

**Table 4:** Comparison of sex hormone levels in patients of observation groups at different times after radiotherapy

		E_2_	*P*_E2_	P_E2_	P	P_P_	P_P_	FSH	P_FSH_	P_FSH_	LH	P_LH_	P_LH_
Postradiation	A	*30.80 ± 20.12*	*1 (Ref.)*		*0.58 ± 0.35*	*1 (Ref.)*		*81.77 ± 27.11*	*1 (Ref.)*		*51.82 ± 17.63*	*1 (Ref.)*	
	B	35.42 ± 18.32	0.58	1 (Ref.)	1.64 ± 0.65	0.719	1 (Ref.)	73.57 ± 27.58	0.492	1 (Ref.)	73.57 ± 27.58	0.071	1 (Ref.)
	C	46.7 ± 37.12	0.249	0.364	0.68 ± 0.19	0.451	0.929	48.70 ± 32.16	0.023	0.065	39.26 ± 13.3	0.008	0.099
3 month after	A	32 ± 16.54	1 (Ref.)		1.06 ± 0.46	1 (Ref.)		97.33 ± 23.29	1 (Ref.)		59.15 ± 15.2	1 (Ref.)	
	B	30.58 ± 8.0	0.795	1 (Ref.)	0.84 ± 0.48	0.094	1 (Ref.)	90.78 ± 31.54	0.593	1 (Ref.)	54.23 ± 19.90	0.528	1 (Ref.)
	C	32.9 ± 18.86	0.911	0.703	0.49 ± 0.25	0.835	0.098	70.19 ± 30.5	0.287	0.137	45.75 ± 20.71	0.115	0.34
6 month after	A	29 ± 14.73	1 (Ref.)		0.49 ± 0.19	1 (Ref.)		88.11 ± 34.43	1 (Ref.)		56.32 ± 24.68	1 (Ref.)	
	B	26.5 ± 10.88	0.652	1 (Ref.)	0.56 ± 0.38	0.566	1 (Ref.)	106.44 ± 37.18	0.598	1 (Ref.)	64.12 ± 16.27	0.841	1 (Ref.)
	C	29.8 ± 7.51	0.88	0.448	0.47 ± 0.32	0.905	0.552	77.81 ± 31.94	0.497	0.269	54.76 ± 24.99	0.89	0.745

E2, estrogen; P, progestin; FSH, follicle‐stimulating hormone; LH, luteinizing hormone; Ref., reference.

### Serum sex hormone levels in the patients of the control group

There was no significant difference between the serum levels of sex hormones in samples collected at different periods of time form the patient of the control group (Table 3, P > 0.05).

### The criteria of ovarian function failure

The standard criteria of ovarian failure include serum concentration of FSH more than 40 U/L and concentration of E_2_ in between 10 and 20 pg/mL. In this study, all patients in the observation groups had serum concentration of FSH higher than 40 U/L, but none of the patients had E_2_ concentration less than 20 pg/mL; therefore, none of the patients was diagnosed with ovarian failure.

## Discussion

In this paper, in order to determine the optimal dose limit to preserve ovarian function in IMRT, we focused on the relationship between ovarian limited dose given to young patients with cervical cancer after ovarian transposition and ovarian function. It has been shown that in the absence of radiation treatment, ovarian endocrine function is well preserved. In all cases studied, radiation after ovarian transposition affected ovarian function; however, the impacts of different radiation therapies were different. There was much less effect on secretion of FSH and LH when the dose was limited to V_10_ < 20%. ROC curve method applied for further analysis yielded optimal ovarian dose limit of V_7.5_ < 26%(*P* = 0.003), with the area under curve 0.740 and 95% CI = 0.606–0.874.

Since 1988, when McCall reported this treatment for patients with cervical cancer who had intact ovaries, it became widely accepted. Therefore, the opportunity to preserve ovaries has become an important reason for young patients to select surgical trials. However, various factors affect ovarian endocrine function, and many studies confirmed that radiotherapy received after ovarian transposition significantly affected ovarian function 3, 6, 7, 8, 9. Ovaries are extremely sensitive to radiation; Chambers et al. reported that radiation dose of 250–300 cGy inhibited ovarian function and radiation dose of 500–1500 cGy induced temporary infertility and transient sex hormone disorder. Furthermore, 2000–3000 cGy of radiation received within a month induced irreversible damage to the ovaries, and resulted in high levels of FSH and LH 10, 11. Buekers et al. reported that in patients who did not receive postoperative radiotherapy, 98% of ovarian function was preserved for as long as 126 month after the procedure, and the average menopause age was 45.8 years. In patients who were given radiotherapy, only 41% of ovarian function was preserved for an average period of 43 month, and the average menopause age was 36.6 years. Previous studies suggested that external pelvic irradiation was the main reason of the lost ovarian function 12. Our results coincide with those obtained by other groups.

For premenopausal young patients with early stages of cervical cancer, some studies reported removing ovaries out of pelvis by ovarian transposition surgery, to keep them away from radiation exposure area. This procedure was followed by irradiation of lymphatic drainage and vaginal residue. This way, ovarian endocrine function can be preserved, which significantly increases the quality of life for young patients who underwent surgery and radiotherapy. Li Sha et al. reported that there was no significant difference in hormone levels of in young patients with cervical cancer after ovarian transposition and postoperative radiotherapy (*P* > 0.05) 13. Zinger et al. evaluated reproductive function of patients who received radical hysterectomy and ovarian transposition. In their study, there was a 22‐year‐old patient with stage Ib cervical cancer, who, 11 years after radical hysterectomy, ovarian transposition, and postoperative radiotherapy, donated two oocytes that were transplanted into surrogate's uterine cavity. This resulted into embryo developments and a full‐term delivery 14. Yet, different results in the relationship between ovarian function and radiotherapy after ovarian transposition in young patients with cervical cancer were obtained in China and abroad 3, 4, 5, 6, 7, 8, 9, 10, 11, 12, 13. The possible reasons for such differences included modifications in the position of ovarian transposition, blood circulation, and ovarian limited dose in radiotherapy.

To conclude, translocation of ovaries higher than 2 cm above the iliac crest and under parietal peritoneum did not completely protect ovaries from the radiation field, and the effect of radiation on endocrine function was still present as suggested by increase in the serum concentration of FSH up to 40 U/L. However, it did not result in ovarian failure, since no decrease in E_2_ level below 10–20 pg/mL was detected. Since all types of radiation treatment affected ovarian function to some degree, limiting radiation dose to V_7.5_ < 26% in IMRT was the preferred option for the preservation of ovarian function. Some limitations to our study include small sample size and short follow‐up time. The holding time of ovarian function needs to be further evaluated in a larger cohort of patients and longer follow‐up time.

## Conflict of Interest

None declared.
